# Prognostic CT features in patients with untreated thymic epithelial tumors

**DOI:** 10.1038/s41598-023-30041-z

**Published:** 2023-02-19

**Authors:** Haiyang Dai, Bowen Lan, Shengkai Li, Yong Huang, Guihua Jiang, Junzhang Tian

**Affiliations:** 1 Department of Medical Imaging, Huizhou Municipal Central Hospital, No. 41, North Eling Road, Huizhou, 516001 People’s Republic of China; 2 Department of Radiology, Shandong Tumor Hospital, No.44, Jiyan Road, Jinan, 250117 People’s Republic of China; 3 Department of Radiology, Guangdong Second Provincial General Hospital, No.466, Xingang Road, Guangzhou, 510317 People’s Republic of China

**Keywords:** Medical research, Risk factors

## Abstract

To determine the prognostic CT features in patients with untreated thymic epithelial tumors (TETs). Clinical data and CT imaging features of 194 patients with pathologically confirmed TETs were retrospectively reviewed. The subjects included 113 male and 81 female patients between 15 and 78 years of age, with a mean age of 53.8 years. Clinical outcomes were categorized according to whether relapse, metastasis or death occurred within 3 years after the first diagnosis. Associations between clinical outcomes and CT imaging features were determined using univariate and multivariate logistic regression analyses, while the survival status was analyzed by Cox regression. In this study, we analyzed 110 thymic carcinomas, 52 high-risk thymomas and 32 low-risk thymomas. Percentages of poor outcome and patient death in thymic carcinomas were much higher than those in patients with high-risk and low-risk thymomas. In the thymic carcinomas groups, 46 patients (41.8%) experienced tumor progression, local relapse or metastasis and were categorized as having poor outcomes; vessel invasion and pericardial mass were confirmed to be independent predictors by logistic regression analysis (p < 0.01). In the high-risk thymoma group, 11 patients (21.2%) were categorized as having poor outcomes, and the CT feature pericardial mass was confirmed to be an independent predictor (p < 0.01). In survival analysis, Cox regression showed that CT features of lung invasion, great vessel invasion, lung metastasis and distant organ metastasis were independent predictors for worse survival in the thymic carcinoma group (p < 0.01), while lung invasion and pericardial mass were independent predictors for worse survival in high-risk thymoma group. No CT features were related to poor outcome and worse survival in the low-risk thymoma group. Patients with thymic carcinoma had poorer prognosis and worse survival than those with high-risk or low-risk thymoma. CT can serve as an important tool for predicting the prognosis and survival of patients with TETs. In this cohort, CT features of vessel invasion and pericardial mass were related to poorer outcomes in those with thymic carcinoma and pericardial mass in those with high-risk thymoma. Features including lung invasion, great vessel invasion, lung metastasis and distant organ metastasis indicate worse survival in thymic carcinoma, whereas lung invasion and pericardial mass indicate worse survival in high-risk thymoma.

## Introduction

Thymic epithelial tumors (TETs) are tumors of epithelial cell origin arising from the thymus gland. TETs account for approximately 20% of mediastinal tumors and 47% of anterior mediastinal tumors, and are the most common tumors in the anterior mediastinum^1,2^. According to the WHO classification, thymic epithelial tumors are classified into thymomas (types A, AB, B1, B2, and B3) and thymic carcinomas, based on the morphology of epithelial cells and the ratio of lymphocyte-to-epithelial cells^3,4^. Although all thymomas have the potential to be invasive, prognosis varies considerably due to the broad range of epithelial malignancies characterized by different histological types and biological behaviors^5^. In general, type A, AB and B1 thymomas are grouped as low-risk thymomas with low invasiveness, whereas type B2 and B3 thymomas are grouped as high-risk thymomas with more aggressiveness^6^. Thymic carcinoma is a rare malignant tumor with high invasiveness and that easily metastasizes. Studies have suggested that the WHO classifications reflects the clinical and prognostic features of TETs^7^, and the majority of studies have demonstrated the clinical correlation of TETs and Masaoka-Koga staging, irrespective of WHO histological categories^8,9^. However, the Masaoka-Koga system is a surgical-pathologic staging system that is applied after surgical resection, and no agreement to date has been reached to define the clinical stage of TETs before surgery. Although empiric evidence has led surgical resection to be the predominant therapy for TETs and complete resection is a consistent prognostic factor^10–12^, some patients with high-risk thymomas and thymic carcinomas rarely to receive complete resection and need multimodal therapy approaches including chemotherapy or radiation therapy (RT)^13^.

CT is the technique of choice for visualization and presurgical characterization of anterior mediastinum lesions^9,14^. The association between CT features and WHO subtypes of TETs has been discussed. Several studies have suggested that CT manifestations, including tumor size, shape, necrosis and lymphadenopathy may help to distinguish pathological TET types^15–17^. Nevertheless, the association between CT imaging features and TET prognosis has not yet been fully assessed. In this study, we retrospectively reviewed the CT findings as well as the prognosis and survival status for 194 patients with pathologically proven TETs in the anterior mediastinum to indentify prognostic CT imaging features.

## Results

### Clinical data and CT imaging features

Among the 194 patients, 32 patients were confirmed of low-risk thymoma (including 7 type A, 17 type AB and 8 type B1), 52 patients were confirmed of high-risk thymoma (including 24 type B2, 11 mixed type B2 and B3, 17 type B3), and 110 patients were confirmed of thymic carcinoma (including 94 squamous cell carcinoma, 8 adenocarcinoma, 3 carcinosarcoma, 3 undifferentiated carcinoma and 2 mucinous basal like carcinoma). The longest diameter (mean ± SD) between thymic carcinomas (7.84 ± 2.37 cm), high-risk thymomas (7.67 ± 3.88 cm) and low-risk thymomas (6.52 ± 3.47 cm) has no statistical significance (F = 2.409, P = 0.093), while the longest diameter in thymic carcinoma was significantly larger than that in low-risk thymoma (t = − 2.031, P = 0.049). The average age of patients with thymic carcinoma (56.8 ± 11.8 years), high-risk thymoma (50.4 ± 13.3 years) and low-risk thymoma (56.4 ± 11.7) was statistically significant (F = 3.909, P = 0.022). Of the 194 patients, 134 received complete or debulking surgical resection; the remaining 60 patients underwent needle biopsy. A total of 113 patients received chemotherapy and radiation therapy; 19 patients received chemotherapy and 30 patients received radiation therapy. The clinical data for the cohort of TETs are illustrated in Table 1.

**Table 1:** Clinical data for 194 cases of thymic epithelial tumors.

	Thymic carcinoma	High-risk thymomoa	Low-risk thymoma	Total
No	110	52	32	194
Male	73	26	14	113
Female	37	26	18	81
Age (year)	56.8 ± 11.8	50.4 ± 13.3	56.4 ± 11.7	
Tumor size (cm)	7.84 ± 2.37	7.67 ± 3.88	6.52 ± 3.47	
Surgical resection	61	41	32	134
Biopsy	49	11	0	60
chemo + RT	94	19	0	113
chemo	14	5	0	19
RT	2	28	0	30
Good outcome	64	41	32	137
Poor outcome	46	11	0	57
Death	32	7	0	39

*chemo* chemo chemotherapy, *RT* radiation therapy.

The CT imaging features had difference between thymic carcinomas, high-risk thymomas and low-risk thymomas. Compared with high-risk thymomas, the presence of irregular shape, heterogeneous enhancement, necrosis, vessel invasion, lymphadenopathy, pericardial effusion and distant organ metastasis were more frequently seen in thymic carcinomas (all P < 0.05), while irregular shape, mediastinal invasion, lung invasion, vessel invasion, pericardial mass, pleural metastasis, lung metastasis, pleural and pericardial effusion were less or rarely seen in low-risk thymomas (all P < 0.05). The main CT features of the 194 patients are summarized in Table 2 and Supplementary Table 1, 2.

**Table 2:** CT features of 194 patients with thymic epithelial tumors.

Characteristics	No. of patients (%)		
	Thymic carcinoma	High-risk thymomoa	Low-risk thymoma
Tumor shape			
Regular	5 (4.5)	15 (28.8)	21 (65.6)
Irregular	105 (95.5)	37 (71.2)	11 (34.4)
Enhancement pattern			
Homogenous	23 (20.9)	20 (38.5)	15 (46.9)
Heterogeneous	87 (79.1)	32 (61.5)	17 (53.1)
Calcification			
Present	44 (40.0)	21 (40.4)	9 (28.1)
Absent	66 (60.0)	31 (59.6)	23 (71.9)
Necrosis			
Present	83 (75.5)	27 (51.9)	14 (43.8)
Absent	27 (24.5)	25 (48.1)	18 (56.2)
Mediastinal invasion			
Present	106 (96.4)	46 (88.5)	4 (12.5)
Absent	4 (3.6)	6 (11.5)	28 (87.5)
Lung invasion			
Present	46 (41.8)	19 (36.5)	0 (0.0)
Absent	64 (58.2)	33 (63.5)	32 (100.0)
Vessel invasion			
Present	39 (35.5)	7 (13.5)	0 (0.0)
Absent	71 (64.5)	45 (86.5)	32 (100.0)
Lymphadenopathy			
Present	67 (60.9)	4 (7.7)	1 (3.1)
Absent	43 (39.1)	48 (92.3)	31 (96.9)
Pericardial mass			
Present	19 (17.3)	9 (17.3)	0 (0.0)
Absent	91 (82.7)	43 (82.7)	32 (100.0)
Pleural metastasis			
Present	27 (24.5)	12 (23.1)	32 (100.0)
Absent	83 (75.5)	40 (76.9)	0 (0.0)
Lung metastasis			
Present	30 (27.3)	7 (13.5)	0 (0.0)
Absent	80 (72.7)	45 (86.5)	32 (100.0)
Distant organ metastasis			
Present	20 (18.2)	1 (1.9)	0 (0.0)
Absent	90 (81.8)	51 (98.1)	32 (100.0)
Pleural effusion			
Present	40 (36.4)	15 (28.8)	0 (0.0)
Absent	70 (63.6)	37 (71.2)	32 (100.0)
Pericardial effusion			
Present	49 (44.5)	8 (15.4)	1 (3.1)
Absent	61 (55.5)	44 (84.6)	31 (96.9)

### Prognostic analysis

**Figure 1:**
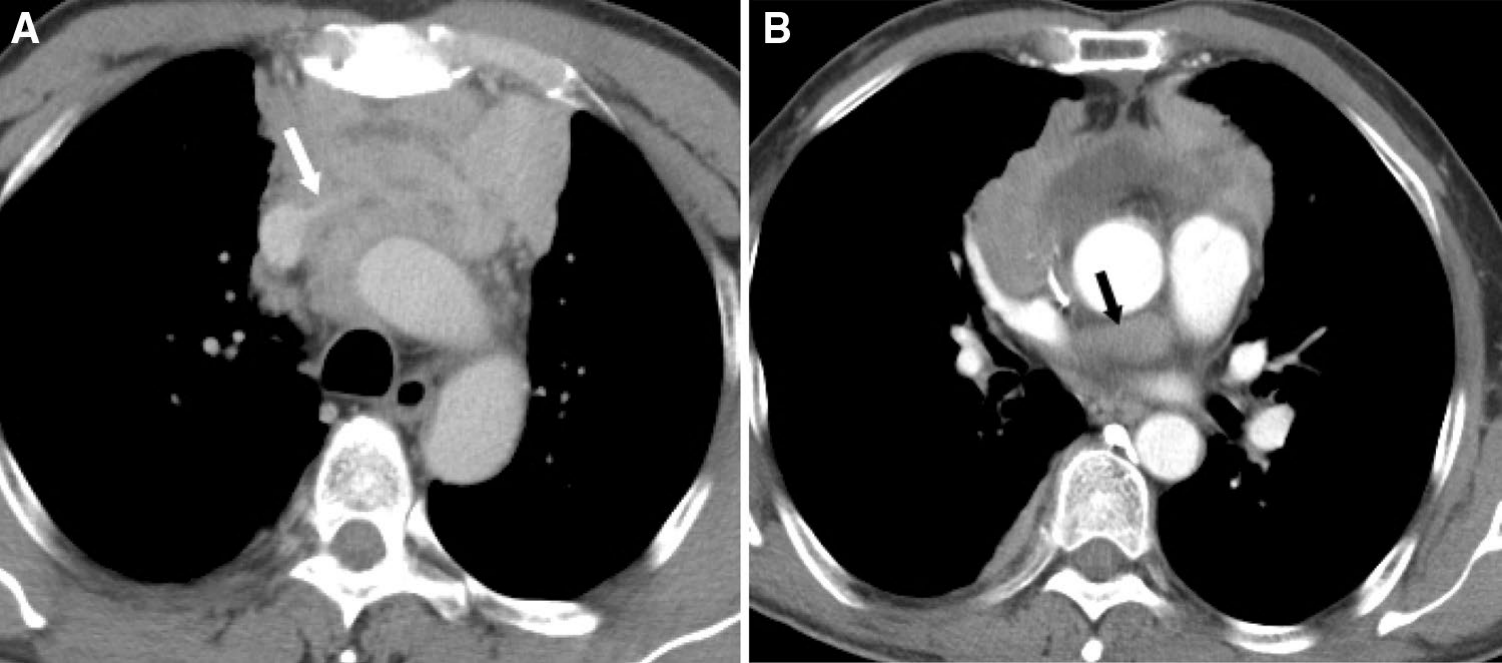
Thymic squamous carcinoma in a 54-year-old man with vessel invasion and pericardial mass. Contrast enhanced CT scan (Axial 5.0 mm) obtained at the level of the aortic arch (**A**) and pulmonary trunk (**B**) shows the interface between the tumor and the left brachiocephalic vein was blurred and the lumen was quite narrowed and compressed (white arrow). A soft tissue mass was seen inside and around the pericardium (black arrow). This patient had tumor progression and died 43.9 months since the first CT examination.

**Figure 2:**
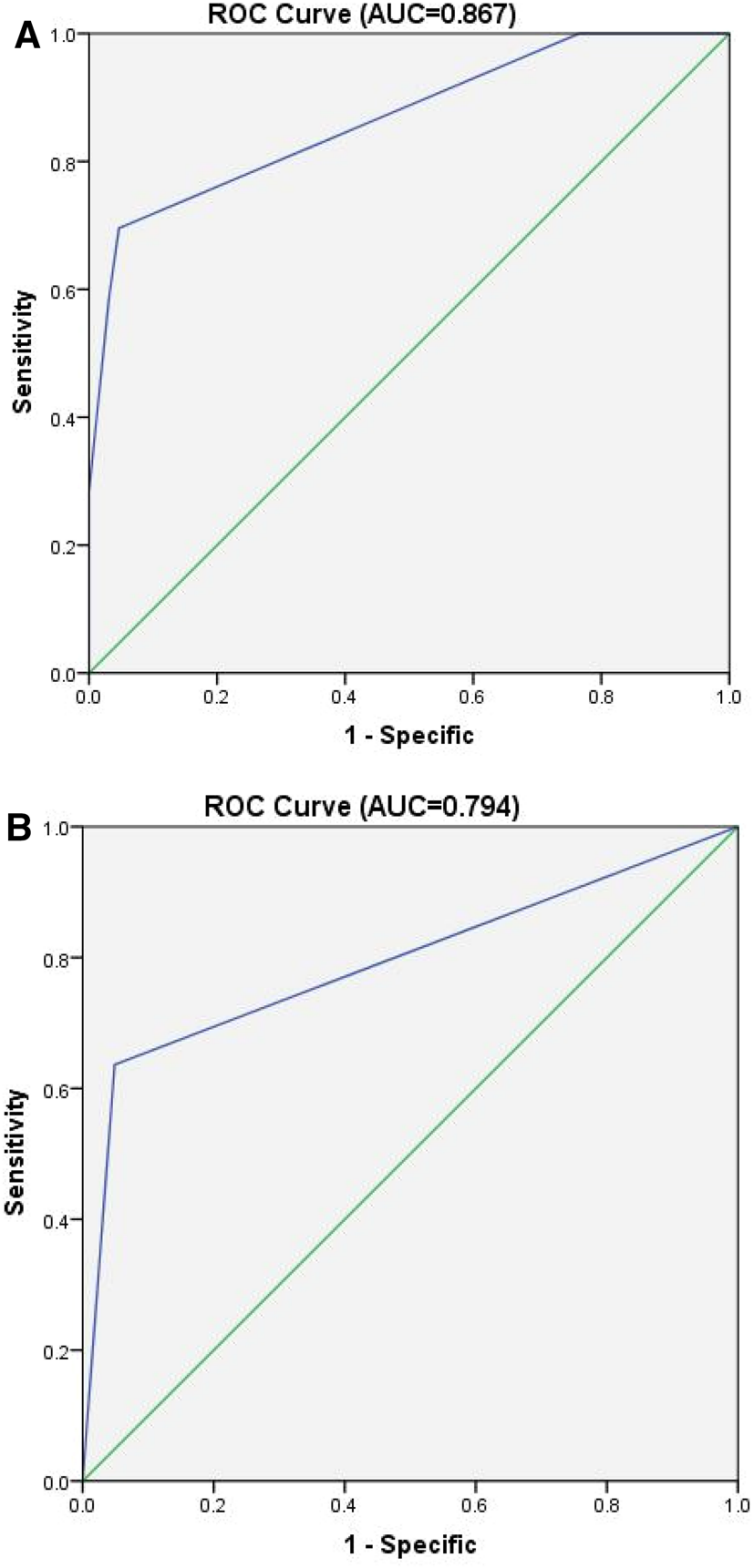
The ROC curve of the multivariate logistical regression model. The area under curve (AUC) was 0.867 and 0.794 for the group of thymic carcinoma (**A**) and high-risk thymoma (**B**), respectively.

In the group of thymic carcinomas, 20 patients had tumor progression, 12 had local relapse and 14 had metastasis within 3 years of the first CT examination. These 46 patients (41.8%) were categorized as the poor outcome group, among whom 32 died. Sixty-four patients (58.2%) survived without any evidence of tumor progression, relapse or metastases since the first CT examination, and were categorized as the good outcome group. In univariate analysis, CT features including enhancement pattern, necrosis, lung invasion, great vessel invasion, pericardial mass, pleural metastasis, lung metastasis, lymphadenopathy, pleural effusion, pericardial effusion and distant organ metastasis were associated with poor clinical outcomes (all P < 0.05, Supplementary Table 3). Multivariate logistical regression analysis showed that only vessel invasion and pericardial mass were significant independent predictors (Table 2, Fig. 1). The presence of vessel invasion and pericardial mass correlated significantly correlated with poor clinical outcome, with OR of 18.61 (P = 0.000) and 8.50 (P = 0.029), respectively. Further ROC curve analysis showed that the area under the curve (AUC) was 0.867 (Fig. 2A), suggesting that the multivariate logistic regression model was a reasonable predictor in this study.

**Figure 3:**
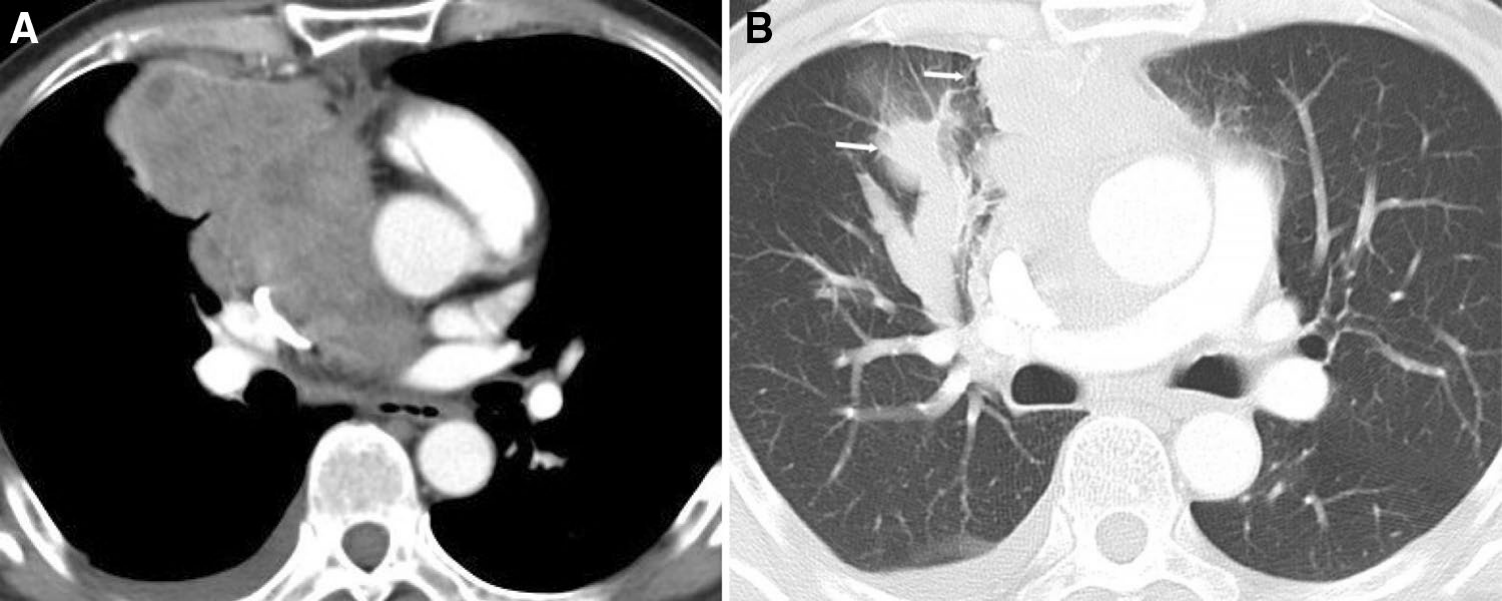
High-risk thymoma (type B2) in a 66-year-old man with pericardial mass and lung invasion. Contrast enhanced CT scan (Axial 5.0 mm) obtained at the level of the aortic root (**A**) and pulmonary trunk (**B**) shows tumor invading the pericardium and form large soft tissue mass inside the pericardium. The interface between the tumor and lung was lost, with compression and effusion in the lung (white arrow). This patient died 23.8 months since the first CT examination.

In the group of high-risk thymomas, 7 patients had tumor progression, 3 had local relapse and 1 had hepatic metastases within 3 years of the first CT examination. These 11 patients (21.1%) were categorized as the poor outcome group, 7 of these patients died. Forty-one patients (78.9%) survived without any evidence of tumor progression, relapse or metastases since the first CT examination and were categorized as the good outcome group. In univariate analysis, CT features including lung invasion, pericardial mass, pleural metastasis, lung metastases, lymphadenopathy, pericardial effusion and pleural effusion were associated with poor clinical outcomes (P < 0.05, Supplementary Table 4); only pericardial mass remained the significantly independent predictor in multivariate logistical regression analysis (Table 3). The presence of pericardial mass correlated significantly with poor clinical outcome (Fig. 3), with an OR of 34.12 (P = 0.000), and the AUC was 0.794 (Fig. 2B), indicating that the multivariate logistic regression model was a reasonable predictor in this study.

**Table 3:** Independent predictors by logistical regression analysis.

Factors	Category	B value	P value	OR (95% CI)
Vessel invasion^a^	Presence	2.924	0.000	18.612 (6.811, 50.862)
Pericardium mass^a^	Presence	2.140	0.029	8.500 (1.247, 57.931)
Pericardial mass^b^	Presence	3.530	0.000	34.125 (5.215, 223.280)

^a^Thymic carcinoma; ^b^high-risk thymoma.

In the group of low-risk thymomas, no patient exhibited any evidence of tumor progression, relapse or metastasis since the first CT examination, and univariate analysis for this group was waived.

### Survival analysis

**Figure 4:**
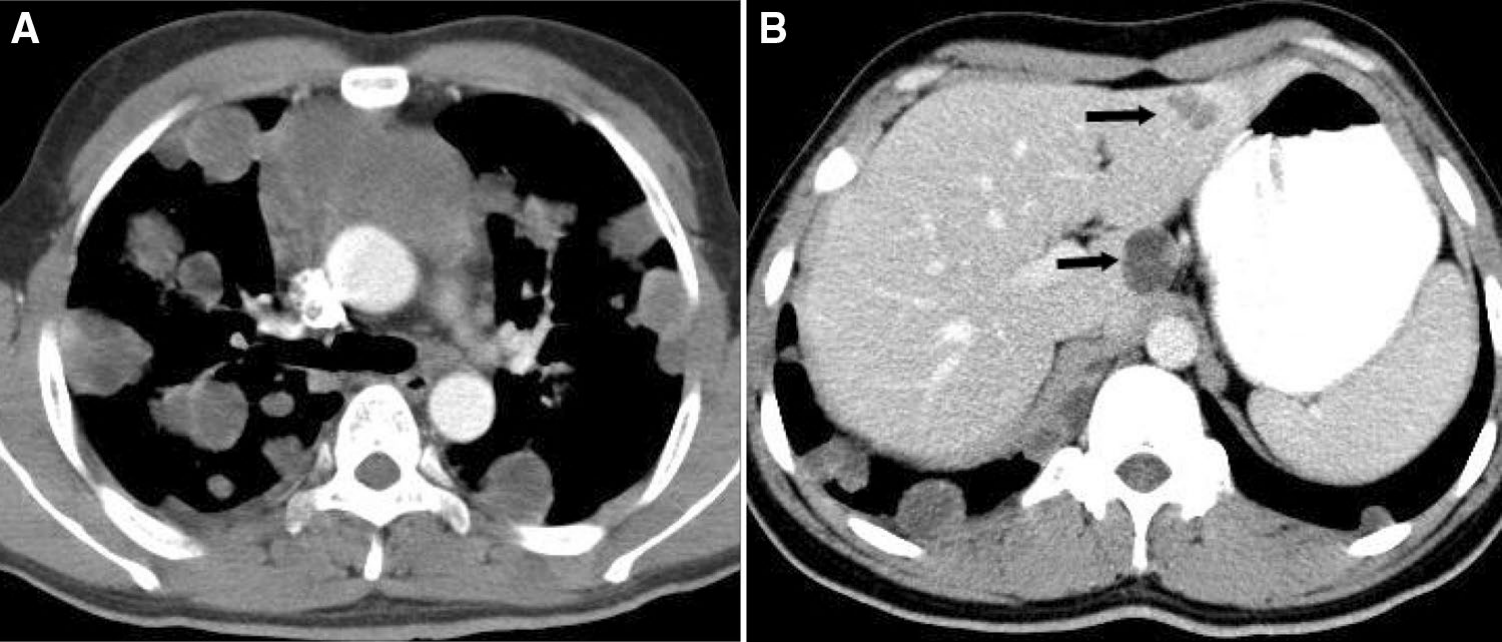
Thymic squamous carcinoma in a 27-year-old man withlung and liver metastasis. Contrast enhanced CT scan (Axial 5.0 mm) obtained at the level of the main bronchi (**A**) and the first porta hepatis (**B**) shows multiple metastasis in the bilateral lung and the left lobe of the liver (black arrow). This patient died 26.1 months since the first CT examination.

In the thymic carcinoma group, 32 patients (29.1%) died during the regular follow-up process, whereas 78 patients (70.9%) were alive by the time of the last follow-up visit. In univariate analysis, CT features including enhancement pattern, necrosis, lung invasion, lung invasion, great vessel invasion, pericardial mass, pleural metastasis, lung metastasis, lymphadenopathy, pleural effusion, pericardial effusion and distant organ metastasis were associated with worse survival status (all P < 0.05, Supplementary Table 5). According to Cox analysis, lung invasion, great vessel invasion, lung metastasis and distant organ metastasis (Fig. 4) were significant independent predictors for worse survival (Table 4).

**Table 4:** Independent predictors by Cox regression analysis for thymic carcinoma.

Factors	Category	B value	*P* value	OR (95% CI)
Lung metastases	Presence	0.912	0.012	2.490 (1.221, 5.080)
Distant metastasis	Presence	1.278	0.001	3.590 (1.657, 7.778)
Lung invasion	Presence	1.439	0.000	4.217 (1.878, 9.473)
Vessel invasion	Presence	0.868	0.025	2.383 (1.115, 5.094)

In the high-risk thymoma group, 7 patients (13.5%) died during follow-up, with 45 patients (86.5%) surviving to the time of the last visit. In univariate analysis, CT features including enhancement pattern, lung invasion, pericardial mass, lung metastasis, pericardial effusion and pleural effusion were associated with worse survival status (all P < 0.05, Supplementary Table 6), but only lung invasion and pericardium mass (Fig. 3) remained significant independent predictors for worse survival in Cox analysis (Table 5).

**Table 5:** Independent predictors by Cox regression analysis for high-risk thymoma.

Factors	Category	B value	P value	OR (95% CI)
Pericardial mass	Presence	4.969	0.012	143.879 (3.029, 6833.33)
Lung invasion	Presence	3.063	0.032	21.386 (1.307, 349.88)

In the low-risk thymoma group, no patients died of thymic tumors by the time of the last follow-up visit. Thus survival analysis was not performed for this group.

## Discussion

It has been widely accepted that the WHO histological classification reflects clinical features and prognostic factors^18,19^. CT and MRI findings of thymic epithelial tumors according to WHO histological criteria have been investigated; however the findings have been interpreted as being of limited value for differentiating various histological subtypes^3,6,20^. Therefore, in the present study we adopted the simplified histological classification introduced by Jeong^21^, who classified the WHO histological classification of TETs into low-risk thymomas (type A, AB and B1), high-risk thymomas (type B2 and B3) and thymic carcinomas. The CT imaging features between low-risk thymomas, high-risk thymomas and thymic carcinomas had widely been discussed. Our study showed that CT features including irregular shape, heterogeneous enhancement, necrosis, vessel invasion, lymphadenopathy, pericardial effusion and distant organ metastasis more frequently occurred in thymic carcinoma than in high-risk thymomas, while irregular shape, mediastinal invasion, lung invasion, vessel invasion, pericardial mass, pleural metastasis, lung metastasis, pleural and pericardial effusion were less or rarely seen in low-risk thymomas, which is consistent with previous studies^15,22,23^.

Studies agree that low-risk thymomas tend to have low aggressiveness and good prognosis but that, high-risk thymomas are more aggressive and thymic carcinoma alone is an independent prognostic factor^5,10,20,24^. As in clinical practice, factors that affect the prognosis of patients are very complex and do not depend on pathology alone. Indeed, the prognosis of patients with the same pathological type may be quite different. It has been widely accepted that the Masaoka-Koga stage and surgical resection are the independent prognostic factors for both overall survival (OS) and recurrence free survival (RFS) in patients with TETs^25–27^. The Masaoka-Koga staging system takes into account tumor invasion, dissemination and metastasis, and is proven to be the most consistent prognostic factor across various studies^28,29^. This can also be asserted for the TNM staging system criteria for thymic malignancies proposed by the International Association for the Study of Lung Cancer (IASLC) and the International Thymic Malignancy Interest Group (ITMIG), which not only describes the extent of tumor invasion but also provides information on lymphatic involvement and tumor dissemination^30–32^. It is commonly accepted that patients with either lower TNM or Masaoka-Koga stage have great chance of receiving complete surgical resection^33,34^. It has been reported that almost all low-risk thymomas and most high-risk thymomas are Masaoka-Koga stage I–II or TNM stage I–II in the clinic, which may explain why these patients have a better prognosis. In the present study, up to 100% of patients with low-risk thymoma and 78.8% of those with high-risk thymoma underwent surgical resection. However for patients with higher TNM or Masaoka-Koga stage, which are more frequently seen in thymic carcinoma and a portion of high-risk thymomas, there is a higher incidence of surrounding structure invasion, such as lung invasion, vessel invasion, pericardial invasion or metastasis, resulting in a lower incidence of complete surgical resection and poor prognosis^27,35,36^. As seen in our study, up to 30% of patients lost the chance of surgical resection at the time of diagnosis, and thus, the Masaoka-Koga or TNM staging data are incomplete. On the other hand, the present data showed that patients who received surgical resection had a higher rate of good prognosis, which is consistent with previous reports^37^. Nevertheless, it should be noted that either the Masaoka-Koga or TNM staging is a pathological stage system confirmed by postoperative pathology. The Masaoka-Koga as well as the TNM staging system did not include tumor size in the definition of the T descriptor, and the TNM system was reported to have limited value in serving as a prognostic prediction model for OS^33,38^.

It was reported that CT provide evidence evaluating capsular, pleural or pericardial invasion, as well as hematogenous or lymphatic dissemination referring to TNM or Masaoka-Koga staging in TETs before surgery^2,14,15^. The results of our study revealed that CT may play an important role in predicting the prognosis and survival status in patients with TETs. CT features, including vessel invasion and pericardial mass in thymic carcinoma, as well as pericardial mass in high-risk thymoma, were found to be independent predictors of poor clinical outcomes. Furthermore, these CT features correlated significantly with tumor progression, relapse or metastasis, as confirmed by regular clinical and CT follow-up. We also found that CT features including lung invasion, great vessel invasion, lung metastasis and distant organ metastasis in thymic carcinoma, as well as lung invasion and pericardium mass in high-risk thymoma, may indicate worse survival. Patients presenting with such features detected by CT would have a higher incidence of potential death. For patients with low-risk thymoma, no CT features were found to be related to poor prognosis or worse survival. From this point of view, it is very important to use CT to identify patients with high prognostic and survival risk factors at an early stage and carry out clinical intervention in an appropriate manner.

Thymic carcinoma is a highly malignant and invasive tumor, and the lung is always the organ most frequently involved^39^. Lung invasion precludes complete surgical resection and may lead to secondary lesions such as pleural effusion and damage to respiratory function, which may result in or accelerate death^40^. It is also commonly accepted that great vessel invasion reduces the chance of complete surgical resection and results in poor prognosis^37,41^. In the present study, both lung invasion and great vessel invasion were confirmed as the independent predictors of poor prognosis and were positively related to patient death in thymic carcinoma, which is consistent with previous studies^23,24^. Lung metastasis and distant organ metastasis, which indicate widespread of tumor cells, were also found to be related to worse survival^42^. Since metastatic tumors may present with more aggressive biological features leading to a lower sensitivity to first-intent systemic treatment compared with locally TETs, the presence of lung and distant organ metastasis is a predictor worse survival, consistent with previous reports^43,44^. It has been found that the imaging features of high-risk thymomas and thymic carcinomas often overlap and are difficult to differentiate from each other. In the present study we found lung invasion and pericardial mass, similar to that of thymic carcinoma, to be independent predictors of poor prognosis and worse survival in patients with high-risk thymoma. Overall, the presence of pericardial mass or lung invasion precludes complete surgical resection and result in poor prognosis.

Our study has several limitations. Firstly, this was a retrospective cohort analysis from two institutions, and several biases, including interdevice and patient selection variability, may exist. In particular, we enrolled patients from a specialized tumor hospital that mainly focuses on complex thymic carcinoma, which is why there were more cases of thymic carcinoma, even though it is a rare malignant tumor in the anterior mediastinal. Secondly, not all patients received surgical resection; hence, the Masaoka-Koga or TNM staging data were not included for analysis. Additionally, the follow-up period might be relatively short for thymic malignancies, in which recurrence beyond 5 years is quite common. Despite these limitations, our study had the strong advantage of confirming important CT features for the prognosis of TETs.

In conclusion, our study confirmed CT plays an important role in predicting the prognosis and survival status of patient with TETs. CT features including vessel invasion and pericardial mass in thymic carcinoma, and pericardium mass in high-risk thymoma are related to poor outcomes. The presence of lung invasion, great vessel invasion, lung metastases and distant organ metastasis in thymic carcinoma, as well as lung invasion and pericardial mass in high-risk thymoma, may be related to worse survival.

## Materials and methods

### Patients

Between May 2009 and June 2020, 194 patients from two tertiary hospitals were enrolled in this retrospective study. The inclusion criteria were as follows: (1) patients underwent surgery or needle biopsy confirmed of thymic epithelial tumors, (2) standard contrast-enhanced CT performed less than 10 days before surgical resection or needle biopsy, and (3) histological pattern available. The exclusion criteria were as follows: (1) any therapy including radiotherapy, chemotherapy or chemoradiotherapy before CT imaging acquisition, (2) unknown histological pattern. This study was approved by the ethics committees of Huizhou Municipal Central Hospital and Shandong Tumor Hospital, and patient informed consent was waived for this retrospective study. All methods were carried out in accordance with relevant guidelines and regulations.

Medical records of all patients were well maintained, and regular follow-up was performed for at least 3 years. Clinical data including sex, age and clinical presentation were reviewed. All patients were Chinese, comprising 113 males and 81 females between 15 and 78 years, with a mean age of 53.8 years. The main symptoms were chest pain (n = 53), cough (n = 41), polypnea (n = 37), myasthenia gravis confirmed by laboratory and physical examination (n = 11), and loss of weight (n = 6); the remaining patients were asymptomatic or discovered incidentally by routine chest radiography. Of the 194 patients, 134 received complete or debulking surgical resection, and the remaining 60 patients received needle biopsy. The pathological results were confirmed by histological and immunohistochemical analyses. The selection of treatment modality was at the clinician’s discretion based on National Comprehensive Cancer Network (NCCN) Clinical Practice Guidelines in Oncology and the consensus based on the Chinese Alliance for Research in Thymomas. All patients received regular clinical and CT follow-up every 3–6 months.

### CT image acquisition

All patients received precontrast and contrast enhanced CT scans before initial treatment. CT examinations were performed using a Siemens 16-slice spiral CT (Somatom sensation 16, Siemens Medical Systems, Erlangen, Germany), GE 64-slice spiral CT (Light Speed VCT, GE Medical Systems, Milwaukee, WI, USA) or Philips 64-slice spiral CT (Brilliance iCT, Philips Healthcare, Cleveland, USA). The imaging parameters included a tube voltage of 120 kV, 200–300 effective mAs, a matrix of 512 × 512 and volume scan with a collimation of 0.625 mm (Siemens and Philips spiral CT) or 0.5 mm (GE 64-slice spiral CT). Contrast-enhanced CT scan was performed with intravenous injection of contrast medium (Iopromide, Bayer-Schering, Berlin, Germany; or Iopamino, Bracco S.P.A., Milan, Italy) by a power injector at a rate of 2.5–3.5 mL/s, with a dosage of 1.5 mL/kg of body weight followed by 20 mL saline flush. When CT scan was complete, all data were transferred to an image workstation where the axial, sagittal and coronal images were reconstructed with a slice thickness of 5 mm.

### CT image analysis

All CT data were reviewed on the Picture Archiving and Communication System (PACS) by two experienced radiologists (Y.H. and B.W.L., both with more than 20 years of experience in diagnostic imaging) independently. The radiologists were blinded to the diagnosis when they evaluated the CT images. Any discrepancies between the two reviewers were resolved by discussion until a consensus was reached. CT imaging features, including tumor size, shape, presence of calcification, enhancement pattern, degree of enhancement, necrosis, mediastinal invasion, lung invasion, vessel invasion, lymphadenopathy, pericardial mass, pericardial effusion, pleural effusion, pleural metastasis, lung metastasis and distant organ metastasis, were determined according to previous report^45^. In particular, tumor size was measured by the longest diameter in the axial, coronal or sagittal dimension. The shape of the tumors was classified as regular or irregular. The regular shape was defined as round or ovoid and irregular shape was lobulated. Calcification was evaluated on precontrast images as present or absent. The pattern of enhancement was recorded as homogenous or heterogeneous. The degree of enhancement was classified as mild when enhancement was less than 20 HU, moderate when 20 HU–40 HU, and marked when greater than 40 HU compared to precontrast scan images. Necrosis was considered when focal areas with low attenuation on precontrast images and no enhancement after contrast medium administration. Mediastinal invasion was considered when the interface between the tumor and mediastinal structures became blurred or not clearly delineated. Lung invasion was considered present when the interface between the tumor and lung was lost, combined with compression and effusion in the lung. Vessel invasion was considered present when the interface between the tumor and blood vessel disappeared combined with compression, gross deformity or a filling defect in the vessel. Lymphadenopathy was considered present if the short-axis diameter of the lymph node was larger than 10 mm, or proven by pathology after surgical resection. Pericardial mass was considered present when a soft tissue mass was seen in the pericardium. Pleural metastasis, lung metastasis and distant organ metastasis were confirmed by needle biopsy or regular follow up. The presence of pleural and pericardial effusion was also assessed.

### Statistical analysis

To determine the prognostic value of radiological parameters, the clinical outcomes of patients were simplified into two categories: a poor outcome if the tumor progresses, relapse or metastasis occurred within 3 years after therapy; a good outcome if the patients survived for more than 3 years with no evidence of tumor progression, relapse or metastasis since the first CT examination. The definition of tumor progression, relapse and metastasis was in accordance with Response Evaluation Criteria in Solid Tumors 1.1 (RECIST 1.1)^46^. A total of 16 CT imaging features were incorporated for analysis in this study, as illustrated in Table 1. Univariate analysis was applied to compare the frequency of these CT imaging features between the poor and good groups using the χ^2^ test. Variables with P < 0.05 were subjected to multivariate logistic regression analysis using a stepwise regression model (forward LR). Odds ratios (OR) as estimates of relative risk with 95% confidence intervals (CI) were obtained for each risk factor. Furthermore, a univariate Cox regression model was used to determine the survival factors for each group. All variables with P < 0.05 in the univariate analysis were included in multivariate Cox analysis using a stepwise regression model (forward LR). All statistical analyses were performed using SPSS software (version 20.0, SPSS Inc., Chicago, IL, USA). A two-sided P < 0.05 was considered statistically significant.

## Supplementary Information

Supplementary Tables.

## Data Availability

All data generated or analyzed during this study are included in this article and supplementary information files.
