# Supplementary material for: Prognostic CT features in patients with untreated thymic epithelial tumors
Source: Sci Rep. 2023 Feb 19;13:2910. doi: 10.1038/s41598-023-30041-z (PMC9939415; doi:10.1038/s41598-023-30041-z)
Supplement: Supplementary file 1 — Supplementary Tables. [file 41598_2023_30041_MOESM1_ESM.docx]

**Prognostic CT features in patients with untreated thymic epithelial tumors**

Haiyang Dai^1*^, Bowen Lan^1^, Shengkai Li^1^,Yong Huang^2^, Guihua Jiang^3^, Junzhang Tian^3^

1. Department of Medical Imaging, Huizhou Municipal Central Hospital, No. 41, North Eling Road, Huizhou 516001, P.R.China

2. Department of Radiology, Shandong Tumor Hospital, No.44,Jiyan Road, Jinan, 250117, P.R. China

3. Department of Radiology, Guangdong Second Provincial General Hospital, No.466, Xingang Road, Guangzhou, 510317, P.R. China

**Supplementary Table 1 CT features between thymic carcinoma and high-risk thymoma**

| Characteristics | Thymic carcinoma | | High-risk thymomoa | | X^2^ | | P | |
| --- | --- | --- | --- | --- | --- | --- | --- | --- |
| Tumor shape |  |  | | 19.268 | | 0.000** | |  |
| Regular | 5 (4.5) | 15 (28.8) | |  | |  | |  |
| Irregular | 105 (95.5) | 37 (71.2) | |  | |  | |  |
| Enhancement pattern |  |  | | 5.579 | | 0.018* | |  |
| Homogenous | 23 (20.9) | 20 (38.5) | |  | |  | |  |
| Heterogeneous | 87 (79.1) | 32 (61.5) | |  | |  | |  |
| Calcification |  |  | | 0.003 | | 0.963 | |  |
| Present | 44 (40.0) | 21 (40.4) | |  | |  | |  |
| Absent | 66 (60.0) | 31 (59.6) | |  | |  | |  |
| Necrosis |  |  | | 8.970 | | 0.003** | |  |
| Present | 83 (75.5) | 27 (51.9) | |  | |  | |  |
| Absent | 27 (24.5) | 25 (48.1) | |  | |  | |  |
| Mediastinal invasion |  |  | |  | |  | |  |
| Present | 106 (96.4) | 46 (88.5) | | Fisher | | 0.077 | |  |
| Absent | 4 (3.6) | 6 (11.5) | |  | |  | |  |
| Lung invasion |  |  | | 0.410 | | 0.522 | |  |
| Present | 46 (41.8) | 19 (36.5) | |  | |  | |  |
| Absent | 64 (58.2) | 33 (63.5) | |  | |  | |  |
| Vessel invasion |  |  | | 8.400 | | 0.004** | |  |
| Present | 39 (35.5) | 7 (13.5) | |  | |  | |  |
| Absent | 71 (64.5) | 45 (86.5) | |  | |  | |  |
| Lymphadenopathy |  |  | | 40.617 | | 0.000** | |  |
| Present | 67 (60.9) | 4 (7.7) | |  | |  | |  |
| Absent | 43 (39.1) | 48 (92.3) | |  | |  | |  |
| Pericardial mass |  |  | | 0.000 | | 0.996 | |  |
| Present | 19 (17.3) | 9 (17.3) | |  | |  | |  |
| Absent | 91 (82.7) | 43 (82.7) | |  | |  | |  |
| Pleural metastasis |  |  | | 0.042 | | 0.838 | |  |
| Present | 27 (24.5) | 12 (23.1) | |  | |  | |  |
| Absent | 83 (75.5) | 40 (76.9) | |  | |  | |  |
| Lung metastasis |  |  | | 3.822 | | 0.051 | |  |
| Present | 30 (27.3) | 7 (13.5) | |  | |  | |  |
| Absent | 80 (72.7) | 45 (86.5) | |  | |  | |  |
| Distant organ metastasis |  |  | | Fisher | | 0.002** | |  |
| Present | 20 (18.2) | 1 (1.9) | |  | |  | |  |
| Absent | 90 (81.8) | 51 (98.1) | |  | |  | |  |
| Pleural effusion |  |  | | 0.890 | | 0.346 | |  |
| Present | 40 (36.4) | 15 (28.8) | |  | |  | |  |
| Absent | 70 (63.6) | 37 (71.2) | |  | |  | |  |
| Pericardial effusion |  |  | | 13.166 | | 0.000** | |  |
| Present | 49 (44.5) | 8 (15.4) | |  | |  | |  |
| Absent | 61 (55.5) | 44 (84.6) | |  | |  | |  |

**Supplementary Table 2 CT features between high-risk and low-risk thymoma**

| Characteristics | High-risk thymomoas | Low-risk thymomas | | X^2^ | P |
| --- | --- | --- | --- | --- | --- |
| Tumor shape |  |  | 10.942 | | 0.001** |
| Regular | 15 (28.8) | 21 (65.6) |  | |  |
| Irregular | 37 (71.2) | 11 (34.4) |  | |  |
| Enhancement pattern |  |  | 0.577 | | 0.448 |
| Homogenous | 20 (38.5) | 15 (46.9) |  | |  |
| Heterogeneous | 32 (61.5) | 17 (53.1) |  | |  |
| Calcification |  |  | 1.297 | | 0.255 |
| Present | 21 (40.4) | 9 (28.1) |  | |  |
| Absent | 31 (59.6) | 23 (71.9) |  | |  |
| Necrosis |  |  | 0.530 | | 0.467 |
| Present | 27 (51.9) | 14 (43.8) |  | |  |
| Absent | 25 (48.1) | 18 (56.2) |  | |  |
| Mediastinal invasion |  |  | Fisher | | 0.000** |
| Present | 46 (88.5) | 4 (12.5) |  | |  |
| Absent | 6 (11.5) | 28 (87.5) |  | |  |
| Lung invasion |  |  | Fisher | | 0.000** |
| Present | 19 (36.5) | 0 (0.0) |  | |  |
| Absent | 33 (63.5) | 32 (100.0) |  | |  |
| Vessel invasion |  |  | Fisher | | 0.041* |
| Present | 7 (13.5) | 0 (0.0) |  | |  |
| Absent | 45 (86.5) | 32 (100.0) |  | |  |
| Lymphadenopathy |  |  | Fisher | | 0.645 |
| Present | 4 (7.7) | 1 (3.1) |  | |  |
| Absent | 48 (92.3) | 31 (96.9) |  | |  |
| Pericardial mass |  |  | Fisher | | 0.012* |
| Present | 9 (17.3) | 0 (0.0) |  | |  |
| Absent | 43 (82.7) | 32 (100.0) |  | |  |
| Pleural metastasis |  |  | Fisher | | 0.000** |
| Present | 12 (23.1) | 32 (100.0) |  | |  |
| Absent | 40 (76.9) | 0 (0.0) |  | |  |
| Lung metastasis |  |  | Fisher | | 0.041* |
| Present | 7 (13.5) | 0 (0.0) |  | |  |
| Absent | 45 (86.5) | 32 (100.0) |  | |  |
| Distant organ metastasis |  |  | Fisher | | 1.000 |
| Present | 1 (1.9) | 0 (0.0) |  | |  |
| Absent | 51 (98.1) | 32 (100.0) |  | |  |
| Pleural effusion |  |  | Fisher | | 0.000** |
| Present | 15 (28.8) | 0 (0.0) |  | |  |
| Absent | 37 (71.2) | 32 (100.0) |  | |  |
| Pericardial effusion |  |  | Fisher | | 0.143 |
| Present | 8 (15.4) | 1 (3.1) |  | |  |
| Absent | 44 (84.6) | 31 (96.9) |  | |  |

**Supplementary Table 3 Clinical outcome of 110 patients with thymic carcinoma**

| Characteristics | Poor outcome | Good outcome | | X^2^ | P |
| --- | --- | --- | --- | --- | --- |
| Tumor shape |  |  | Fisher | | 0.127 |
| Regular | 41 (89.1) | 62 (96.9) |  | |  |
| Irregular | 5 (10.9) | 2 (3.1) |  | |  |
| Enhancement pattern |  |  | 7.132 | | 0.008** |
| Homogenous | 4 (8.7) | 19 (29.7) |  | |  |
| Heterogeneous | 42 (91.3) | 45 (70.3) |  | |  |
| Calcification |  |  | 1.052 | | 0.305 |
| Present | 21 (45.7) | 23 (35.9) |  | |  |
| Absent | 25 (54.3) | 41 (64.1) |  | |  |
| Necrosis |  |  | 7.984 | | 0.005** |
| Present | 41 (89.1) | 42 (65.6) |  | |  |
| Absent | 5 (10.9) | 22 (34.4) |  | |  |
| Mediastinal invasion |  |  | Fisher | | 0.138 |
| Present | 46 (100.0) | 60 (93.8) |  | |  |
| Absent | 0 (0.0) | 4 (6.2) |  | |  |
| Lung invasion |  |  | 14.639 | | 0.000** |
| Present | 29 (63.0) | 17 (26.6) |  | |  |
| Absent | 17 (37.0) | 47 (73.4) |  | |  |
| Vessel invasion |  |  | 40.199 | | 0.000** |
| Present | 32 (69.6) | 7 (10.9) |  | |  |
| Absent | 14 (30.4) | 57 (89.1) |  | |  |
| Lymphadenopathy |  |  | 5.615 | | 0.018* |
| Present | 34 (73.9) | 33 (51.6) |  | |  |
| Absent | 12 (26.1) | 31 (48.4) |  | |  |
| Pericardial mass |  |  | Fisher | | 0.000** |
| Present | 17 (37.0) | 2 (3.1) |  | |  |
| Absent | 29 (63.0) | 62 (96.9) |  | |  |
| Pleural metastasis |  |  | 15.302 | | 0.000** |
| Present | 20 (43.5) | 7 (10.9) |  | |  |
| Absent | 26 (56.5) | 57 (89.1) |  | |  |
| Lung metastasis |  |  | 7.848 | | 0.005** |
| Present | 19 (41.3) | 11 (17.2) |  | |  |
| Absent | 27 (58.7) | 53 (82.8) |  | |  |
| Distant organ metastasis |  |  | 7.979 | | 0.005** |
| Present | 14 (30.4) | 6 (9.4) |  | |  |
| Absent | 32 (69.6) | 58 (90.6) |  | |  |
| Pleural effusion |  |  | 4.489 | | 0.034* |
| Present | 22 (47.8) | 18 (28.1) |  | |  |
| Absent | 24 (52.2) | 46 (71.9) |  | |  |
| Pericardial effusion |  |  | 8.529 | | 0.003** |
| Present | 28 (60.9) | 21 (32.8) |  | |  |
| Absent | 18 (39.1) | 43 (67.2) |  | |  |

**Supplementary Table 4 Clinical outcome of 52 patients with high-risk thymoma**

| Characteristics | Poor outcome | Good outcome | | X^2^ | P |
| --- | --- | --- | --- | --- | --- |
| Tumor shape |  |  | Fisher | | 0.000 |
| Regular | 1 (9.1) | 40 (97.6) |  | |  |
| Irregular | 10 (90.9) | 1 (2.4) |  | |  |
| Enhancement pattern |  |  | Fisher | | 0.170 |
| Homogenous | 2 (18.2) | 18 (43.9) |  | |  |
| Heterogeneous | 9 (81.8) | 23 (56.1) |  | |  |
| Calcification |  |  | 0.149 | | 0.700 |
| Present | 5 (45.5) | 16 (39.0) |  | |  |
| Absent | 6 (54.5) | 25 (61.0) |  | |  |
| Necrosis |  |  | Fisher | | 0.177 |
| Present | 8 (72.7) | 19 (46.3) |  | |  |
| Absent | 3 (27.3) | 22 (53.7) |  | |  |
| Mediastinal invasion |  |  | Fisher | | 0.322 |
| Present | 11 (100.0) | 35 (85.4) |  | |  |
| Absent | 0 (0.0) | 6 (14.6) |  | |  |
| Lung invasion |  |  | Fisher | | 0.011* |
| Present | 8 (72.7) | 11 (26.8) |  | |  |
| Absent | 3 (27.3) | 30(73.2) |  | |  |
| Vessel invasion |  |  | Fisher | | 0.154 |
| Present | 3 (27.3) | 4 (9.8) |  | |  |
| Absent | 8 (72.7) | 37 (90.2) |  | |  |
| Lymphadenopathy |  |  | Fisher | | 0.026* |
| Present | 3 (27.3) | 1 (2.4) |  | |  |
| Absent | 8 (72.7) | 40 (97.6) |  | |  |
| Pericardial mass |  |  | Fisher | | 0.000** |
| Present | 7 (63.6) | 2 (4.9) |  | |  |
| Absent | 4 (36.4) | 39 (95.1) |  | |  |
| Pleural metastasis |  |  | 7.783 | | 0.005** |
| Present | 6 (54.5) | 6 (14.6) |  | |  |
| Absent | 5 (45.5) | 35 (85.4) |  | |  |
| Lung metastasis |  |  | Fisher | | 0.029* |
| Present | 4(36.4) | 3 (7.3) |  | |  |
| Absent | 7 (63.6) | 38 (92.7) |  | |  |
| Distant organ metastasis |  |  | Fisher | | 0.212 |
| Present | 1 (9.1) | 0 (0.0) |  | |  |
| Absent | 10 (90.9) | 41 (100.0) |  | |  |
| Pleural effusion |  |  | Fisher | | 0.001** |
| Present | 8 (72.7) | 7 (17.1) |  | |  |
| Absent | 3 (27.3) | 34 (82.9) |  | |  |
| Pericardial effusion |  |  | Fisher | | 0.007** |
| Present | 5 (45.5) | 3 (7.3) |  | |  |
| Absent | 6 (54.5) | 38 (92.7) |  | |  |

**Supplementary Table 5 Survival status of 110 patients with thymic carcinoma**

| Characteristics | Dead | Alive | | X^2^ | P |
| --- | --- | --- | --- | --- | --- |
| Tumor shape |  |  | Fisher | | 0.319 |
| Regular | 0 (0.0) | 5 (6.4) |  | |  |
| Irregular | 32 (100.0) | 73 (93.6) |  | |  |
| Enhancement pattern |  |  | Fisher | | 0.019* |
| Homogenous | 2 (6.2) | 21 (26.9) |  | |  |
| Heterogeneous | 30 (93.8) | 57 (73.1) |  | |  |
| Calcification |  |  | 3.239 | | 0.072 |
| Present | 17 (53.1) | 27 (34.6) |  | |  |
| Absent | 15 (46.9) | 51 (65.4) |  | |  |
| Necrosis |  |  | Fisher | | 0.003** |
| Present | 30 (93.8) | 53 (67.9) |  | |  |
| Absent | 2 (6.2) | 25 (32.1) |  | |  |
| Mediastinal invasion |  |  | Fisher | | 0.320 |
| Present | 32 (100.0) | 74 (94.9) |  | |  |
| Absent | 0 (0.0) | 4 (5.1) |  | |  |
| Lung invasion |  |  | 16.756 | | 0.000** |
| Present | 23 (71.9) | 23 (29.5) |  | |  |
| Absent | 9 (28.1) | 55 (70.5) |  | |  |
| Vessel invasion |  |  | 20.455 | | 0.000** |
| Present | 22 (68.8) | 18 (23.1) |  | |  |
| Absent | 10 (31.2) | 60 (76.9) |  | |  |
| Lymphadenopathy |  |  | 7.84 | | 0.005** |
| Present | 26 (81.2) | 41 (52.6) |  | |  |
| Absent | 6 (18.8) | 37 (47.4) |  | |  |
| Pericardial mass |  |  | 12.922 | | 0.000** |
| Present | 12 (37.5) | 7 (9.0) |  | |  |
| Absent | 20 (62.5) | 71 (91.0) |  | |  |
| Pleural metastasis |  |  | 12.149 | | 0.000** |
| Present | 15 (46.9) | 12 (15.4) |  | |  |
| Absent | 17 (53.1) | 66 (84.6) |  | |  |
| Lung metastasis |  |  | 8.742 | | 0.003** |
| Present | 15 (46.9) | 15 (19.2) |  | |  |
| Absent | 17 (53.1) | 63 (80.8) |  | |  |
| Distant organ metastasis |  |  | 7.955 | | 0.005** |
| Present | 11 (34.4) | 9 (11.5) |  | |  |
| Absent | 21 (65.6) | 69 (88.5) |  | |  |
| Pleural effusion |  |  | 10.327 | | 0.001* |
| Present | 19 (59.4) | 21 (26.9) |  | |  |
| Absent | 13 (40.6) | 57 (73.1) |  | |  |
| Pericardial effusion |  |  | 5.889 | | 0.015* |
| Present | 20 (62.5) | 29 (37.2) |  | |  |
| Absent | 12 (37.5) | 49 (62.8) |  | |  |

**Supplementary Table 6 Survival status of 52 patients with high-risk thymoma**

| Characteristics | Dead | Alive | | X^2^ | P |
| --- | --- | --- | --- | --- | --- |
| Tumor shape |  |  | Fisher | | 0.658 |
| Regular | 1 (14.3) | 14 (31.1) |  | |  |
| Irregular | 6 (85.7) | 31 (68.9) |  | |  |
| Enhancement pattern |  |  | Fisher | | 0.035* |
| Homogenous | 0 (0.0) | 20 (44.4) |  | |  |
| Heterogeneous | 7 (100.0) | 25 (55.6) |  | |  |
| Calcification |  |  | Fisher | | 0.420 |
| Present | 4 (57.1) | 17 (37.8) |  | |  |
| Absent | 3 (42.9) | 28 (62.2) |  | |  |
| Necrosis |  |  | Fisher | | 0.422 |
| Present | 5 (71.4) | 22 (48.9) |  | |  |
| Absent | 2 (28.6) | 23 (51.1) |  | |  |
| Mediastinal invasion |  |  | Fisher | | 0.580 |
| Present | 7 (100.0) | 39 (86.7) |  | |  |
| Absent | 0 (0.0) | 6 (13.3) |  | |  |
| Lung invasion |  |  | Fisher | | 0.007** |
| Present | 6 (85.7) | 13 (28.9) |  | |  |
| Absent | 1 (14.3) | 32 (71.1) |  | |  |
| Vessel invasion |  |  | Fisher | | 0.235 |
| Present | 2 (28.6) | 5 (11.1) |  | |  |
| Absent | 5 (71.4) | 40 (88.9) |  | |  |
| Lymphadenopathy |  |  | Fisher | | 0.083 |
| Present | 2 (28.6) | 2 (4.4) |  | |  |
| Absent | 5 (71.4) | 43 (95.6) |  | |  |
| Pericardial mass |  |  | Fisher | | 0.000** |
| Present | 6 (85.7) | 3 (6.7) |  | |  |
| Absent | 1 (14.3) | 42 (93.3) |  | |  |
| Pleural metastasis |  |  | Fisher | | 0.331 |
| Present | 3 (42.9) | 9 (20.0) |  | |  |
| Absent | 4 (57.1) | 36 (80.0) |  | |  |
| Lung metastasis |  |  | Fisher | | 0.026* |
| Present | 3 (42.9) | 3 (6.7) |  | |  |
| Absent | 4 (57.1) | 42 (93.3) |  | |  |
| Distant organ metastasis |  |  | Fisher | | 0.135 |
| Present | 1 (14.3) | 0 (0.0) |  | |  |
| Absent | 6 (85.7) | 45 (100.0) |  | |  |
| Pleural effusion |  |  | Fisher | | 0.001** |
| Present | 6 (85.7) | 9 (20.0) |  | |  |
| Absent | 1 (14.3) | 36 (80.0) |  | |  |
| Pericardial effusion |  |  | Fisher | | 0.000** |
| Present | 5 (71.4) | 3 (6.7) |  | |  |
| Absent | 2 (28.6) | 42 (93.3) |  | |  |

*:P<0.05; **：P<0.01
